# Characterization of Bioactive Compounds of *Opuntia ficus-indica* (L.) Mill. Seeds from Spanish Cultivars

**DOI:** 10.3390/molecules25235734

**Published:** 2020-12-04

**Authors:** Joanna Kolniak-Ostek, Agnieszka Kita, Joanna Miedzianka, Lucia Andreu-Coll, Pilar Legua, Francisca Hernandez

**Affiliations:** 1 Department of Fruit, Vegetable and Plant Nutraceutical Technology, Wroclaw University of Environmental and Life Sciences, Chelmonskiego 37 Street, 51-630 Wroclaw, Poland; 2 Department of Food Storage and Technology, Wroclaw University of Environmental and Life Sciences, Chelmonskiego 37 Street, 51-630 Wroclaw, Poland; agnieszka.kita@upwr.edu.pl (A.K.); joanna.miedzianka@upwr.edu.pl (J.M.); 3 Department of Plant Sciences and Microbiology, Research Group “Plant Production and Technology”, Polytechnic School of Orihuela, Miguel Hernández University of Elche (UMH), Carretera de Beniel, Km. 3.2, 03312 Orihuela, Alicante, Spain; lucia.andreu1@gmail.com (L.A.-C.); p.legua@umh.es (P.L.); francisca.hernandez@umh.es (F.H.)

**Keywords:** prickly pear, UPLC-MS, phenolic compounds, fatty acids, amino acids

## Abstract

*Opuntia ficus-indica* (L.) Mill. is the Cactaceae plant with the greatest economic relevance in the world. It can be used for medicinal purposes, animal nutrition, production of biofuels and phytoremediation of soils. Due to its high content of bioactive compounds, the prickly pear has antioxidant, antimicrobial and anticancer properties. The aim of this study was to determine the polyphenolic, fatty acid and amino acid profile and characterize the antioxidant capacity of seeds of seven Spanish prickly pear cultivars. A total of 21 metabolites, mainly phenolic acids and flavonols, were identified using ultraperformance liquid chromatography photodiode detector quadrupole/time-of-flight mass spectrometry (UPLC-PDA-Q/TOF-MS). Significant differences were found in the phenolic concentrations of the investigated varieties. The highest amount of phenolic compounds (266.67 mg/kg dry matter) were found in the “Nopal espinoso” variety, while the “Fresa” variety was characterized by the lowest content (34.07 mg/kg DM) of these compounds. In vitro antioxidant capacity was positively correlated with the amount of polyphenols. The amino acid composition of protein contained in prickly pear seeds was influenced by the variety. Glutamic acid was the predominant amino acid followed by arginine, aspartic acid and leucine, independent of prickly pear variety. Overall, 13 different fatty acids were identified and assessed in prickly pear seeds. The dominant fatty acid was linoleic acid, with content varying between 57.72% “Nopal ovalado” and 63.11% “Nopal espinoso”.

## 1. Introduction

Commonly known as the prickly pear or cactus pear, *Opuntia ficus-indica* (L.) Mill. is the Cactaceae plant with the greatest economic relevance in the world. This plant is mainly known for its fruit, but cladodes are also consumed, mainly in Mexico, which is the country with the largest area under cultivation and also the largest producer [1,2]. Both are consumed fresh, but can also be consumed cooked, canned, dehydrated and as concentrated juice, jams and syrups, among other forms [1,3]. Besides that, the prickly pear has been used for medicinal purposes, animal nutrition, the production of biofuels and phytoremediation of soils, among others [4,5].

The pulp is the edible part of prickly pear fruit and is mainly composed of water (84–90%) and reducing sugars, mainly glucose and fructose (10–15%) [6,7]. The fruit contains a large number of seeds, about 0.24 g/g, constituting about 10–15% of the edible pulp and 30–40% on a dry weight basis [1,7,8]. An edible oil can be obtained from prickly pear seeds, which is rich in polyunsaturated and monounsaturated fatty acids, of which linoleic acid is the predominant fatty acid, followed by oleic acid [9,10,11]. The consumption of these kinds of fatty acids is related to health benefits and contributes to the improvement of various health conditions such as cardiovascular diseases, obesity and diabetes mellitus, among others [12].

Antioxidant activity is one of the major mechanisms by which fruit and vegetables provide health benefits. The high amounts of polyphenols, which show strong antioxidative properties attributed to their ability to scavenge free radicals and to chelate metal ions involved in their production, contribute to the strong antioxidant activity of prickly pear seeds [8,13]. Besides that, prickly pear seeds contain 11–17% protein, higher than the content in fruit peel and pulp, glutamic acid, aspartic acid, arginine and glycine predominating in its amino acid profile, and are also rich in minerals [11,14,15]. However, the composition of prickly pear seeds can vary among cultivars, varieties and crop environmental factors, among others [16].

The seeds of the prickly pear are usually discarded after the extraction of pulp, providing a large amount of seeds as waste. The study of the composition of these seeds could help to find possible uses in the cosmetic and pharmaceutical industries, animal feed, and also in the human diet as a new source of oil and meal. This work was carried out on the seeds of seven Spanish prickly pear cultivars. The main objectives of this comparative study were (i) to quantify the antioxidant activity and phenolic compounds in these seeds, (ii) to determine the fatty acid profile and (iii) to define the amino acid profile. This research intends to value prickly pears of local origin; the results will provide specific information about the composition of prickly pear seeds, and could be valuable to the food, cosmetic and pharmaceutic industries in order to utilize this byproduct. Besides its health-promoting properties, the prickly pear is also a very profitable crop in Spain, in addition to contributing to the mitigation of climate change in arid and semiarid regions by sequestration. The study of the composition of these cultivars constitutes an advance in the knowledge of their properties and in the elaboration of derived products. Due to the exploitation of the juice, a large amount of waste is left, including the seeds, currently used in animal feed, this work evaluates the composition of the seeds for use as a food supplement and the possibility of them being used in the cosmetic and pharmaceutical industries.

## 2. Results and Discussion

### 2.1. Metabolite Identification Using UPLC-MS Analysis

**Figure 1:**
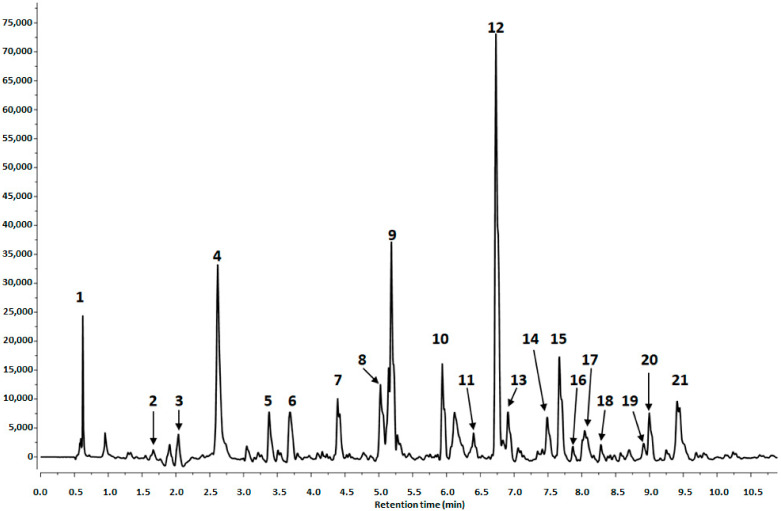
Ultraperformance liquid chromatography (UPLC)–MS chromatogram profile of the “Orito” cultivar at 280 nm. Peak number identities are displayed in Table 1.

The secondary metabolites of *Opuntia ficus-indica* (L.) Mill extracts were determined using an ACQUITY UPLC system equipped with a PDA detector and G2 Q-Tof micromass spectrometer (Waters, Manchester, UK) operating in negative mode. Figure 1 shows the LC-DAD chromatogram of the “Orito” cultivar. Qualitative analysis results with their UV and mass spectral data are summarized in Table 1.

Two major classes of phenolic compounds were identified—phenolic acids and flavonols. In addition, two organic acids were found and identified.

#### 2.1.1. Phenolic Acid Derivatives

Four derivatives of ferulic acid (peaks 6, 9, 11 and 13), two derivatives of caffeic acid (peaks 8 and 10) and one derivative each of protocatechuic acid (peak 5), piscidic acid (peak 4) and eucomic acid (peak 7) were identified in prickly pear seeds.

Peaks 6, 9, 11 and 13 showed a similar fragmentation pattern with product ions at *m*/*z* 193 and 175 [M − H − 18]^−^, corresponding to the loss of a ferulic acid moiety and suggesting that these metabolites are ferulic acid derivatives [17].

In the group of caffeic acid derivatives, two caffeic acid hexoses were detected. Peaks 8 and 10 had pseudomolecular ions at *m*/*z* 341.0837 and 341.0697, respectively, and fragmentation ions at *m*/*z* 179 which correspond to the loss of hexose residues (162u).

Peak number 4 showed a molecular ion [M − H]^−^ at *m*/*z* 255.0366 with product ions at *m*/*z* 237 [M − H − 18]^−^, 193 [M − H − 62]^−^ and 165 [M − H − 90]^−^, corresponding to the loss of two water, carbon dioxide and carbon oxide residues, and was identified as piscidic acid [18].

Peak number 5 with a pseudomolecular ion at *m*/*z* 315.0781 and pseudomolecular ion at *m*/*z* 153, which corresponded to the loss of a hexoside residue (162u), was identified as protocatechuic acid hexoside [19].

Peak number 7 showed a molecular ion at *m*/*z* 239.0416 and product ions at *m*/*z* 179 [M − H − 60]^−^ and 149 [M − H − 90]^−^, and was identified as eucomic acid according to the literature [20].

Phenolic acids and their derivatives have previously been identified in prickly pear fruits and juices. For example, Faraq et al. [20] in their study on three *Opuntia ficus indica* fruit cultivars have identified derivatives of caffeic and ferulic acids. Ferulic and protocatechuic acids have been identified by Guevara-Figueroa et al. [21] in their study on prickly pear cladodes, while Mata et al. [22] have identified among others piscidic and eucomic acids in *Opuntia ficus-indica* juices. Up to now, only ferulic acid had been identified in prickly pear seeds [8], while piscidic, eucomic, protocatechuic and caffeic acid and their derivatives have now been identified in seeds for the first time.

#### 2.1.2. Flavonols

Eight flavonols were detected in prickly pear seed extracts, comprising six isorhamnetin derivatives (peaks 16–21) and three quercetin derivatives (peaks 3, 14 and 15) (Table 1).

The quercetin derivatives were quercetin aglycone, quercetin 3-*O*-rutinoside (rutin), and quercetin-3-*O*-galactoside. Each of the compounds has the typical quercetin fragment at *m*/*z* 301. Peak 3 with a molecular ion [M − H]^−^ at *m*/*z* 301.0920, was identified as quercetin. Peak 14, with a pseudomolecular ion at *m*/*z* 609.1295, was identified as a quercetin 3-*O*-rutinoside (rutin), and peak 15 with a molecular ion at *m*/*z* 463.1399, was identified as quercetin-3-*O*-galactoside. Quercetin 3-*O*-rutinoside (rutin) and 3-*O*-galactoside are commonly present flavonoids in plants, which have been detected previously, for example, in methanol extracts from the thornless form of Tunisian *O. ficus-indica* [23,24]. Quercetin derivatives have previously been identified in prickly pear fruit (peel and flesh) [20,23] and in its juices [22] and flowers [24], but have not been studied previously in the seeds of this plant.

In the group of isorhamnetin derivatives, isorhamnetin-pentosyl rutinoside (peak 16), -pentosyl rhamnoside (peak 17), -3-*O*-rutinoside (peak 18), -3-*O*-galactoside (peak 19), -3-*O*-glucoside (peak 20) and -acylated-hexoside (peak 21) were found. All of them possess the typical isorhamnetin fragment at *m/z* 315 formed by the cleavage of the hexoside residues, i.e., -galactoside (-162u), -rutinoside (-308u) and -acylated-hexosides (-162u-42u), from the isorhamnetin glycosides. Isorhamnetin derivatives are commonly present in various species of prickly pear. They can be found in flowers [24,25], pulp and peel [23]. Isorhamnetin derivatives have also been detected both in the juice [22] and methanolic extracts of *O. ficus-indica* [20], however, they have not been identified in prickly pear seeds.

#### 2.1.3. Organic Acids

Two organic acids—gluconic and (iso)citric acid—were identified in the seeds of the prickly pear (Table 1). Peak 1, with a molecular ion [M − H]^−^ at *m*/*z* 195.0522, and a typical fragmentation pattern with product ions at *m*/*z* 177 and 159 corresponding to the loss of two water residues (-18u and -36u), was identified as gluconic acid [26]. Peak 2 had a pseudomolecular ion at *m*/*z* 191.0051 and a product ion at *m*/*z* 11.9974 and was identified as (iso)citric acid. Gluconic and (iso)citric acids have previously been identified in *O.ficus indica* fruit extracts [20]. However, these compounds have not been identified in prickly pear seeds.

#### 2.1.4. Other Compounds

Peak 12 had pseudomolecular ion at *m*/*z* 565.1764, and fragmentation ions at *m*/*z* 339.1087 and 327.1086, which corresponded to the loss of 226u and 238u, and was a major peak in prickly pear seeds (Table 1, Figure 1). This compound has previously been detected in *Opuntia ficus-indica* fruit [20], but as in our case, it was not identified.

### 2.2. Quantitative Analysis of Polyphenols

Quantitative analysis of prickly pear seeds was conducted by external calibration curves using selected reference compounds (Materials and Methods: Section 3.3). The concentration of the individual substances was expressed as mg/kg dry matter (DM) (Table 2).

The analysis showed differences in the content of polyphenols between the tested cultivars. The highest concentration of phenolic acids and flavonols (171.60 and 95.07 mg/kg DM, respectively) was determined in “Nopal espinoso” cultivar (Table 2). “Fresa” cultivar was characterized by the lowest concentration of both polyphenolic groups (19.05 and 34.07 mg/kg DM, respectively). In all samples tested, phenolic acids were the dominant group of phenolic compounds as compared to flavonols, and their total amount was 17% higher.

These results are in agreement with the results presented by Guevara-Figueroa et al. [21], who analyzed the concentration of phenolic compounds in commercial and wild prickly pear cladodes. De Wit et al. [27] obtained slightly higher values, ranging from 74.86 mg/kg to 291.46 mg/kg for seeds from 8 different cultivars of prickly pear. These differences may be due to cultivar and genetic factors, growth conditions, as well as harvesting time, degree of ripeness or fruit processing, and above all, the determination methods [27].

The results obtained show that the proportion and concentration of phenolic compounds in plants are dependent on the anatomical part. The variability of phenolics in plant tissues depends on many factors, such as temperature, UV light and nutrition [28,29,30].

### 2.3. In Vitro Antioxidant Activity

The in vitro antioxidant activity of *O. ficus-indica* seeds was measured as the ferric reducing capacity by the FRAP method and free radical scavenging activity (DPPH and ABTS methods) and is listed in Table 3. The results of the DPPH, ABTS and FRAP methods were expressed in the same units, i.e., mmol of Trolox equivalent per kg of prickly pear DM.

The highest in vitro antioxidant activity determined by DPPH, ABTS and FRAP methods was observed in the “Nopal espinoso” variety (4.99, 11.67 and 15.64 mmol Trolox/kg DM, respectively), while the “Fresa” variety was characterized by the lowest results—1.39, 7.08 and 3.67 mmol Trolox/kg DM, respectively. Our results were slightly lower than those reported by other authors. Andreu et al. [6] reported that ABTS in vitro antioxidant capacity of prickly pear cladode and fruit was 18.8 mmol Trolox/kg (dw) and 26.8 mmol Trolox/kg (dw), respectively, DPPH in vitro activity was 17.4 mmol Trolox/kg (dw) and 58.0 mmol Trolox/kg (dw), respectively, while FRAP in vitro capacity for cladode and fruit was 85.3 mmol Trolox/kg (dw) and 40.2 mmol Trolox/kg (dw), respectively. These differences may be due to anatomical part of the prickly pear examined. Literature data [31,32] indicate that polyphenols play an important role in antioxidant activity, in particular scavenging DPPH. The content and proportion of phenolic compounds in plants are closely related to the anatomical part. The in vitro antioxidant capacity of the tested seeds was positively correlated with the amount of polyphenolic substances. The results obtained show a high correlation coefficient between the content of polyphenolic compounds and in vitro antioxidant capacity determined by the DPPH, ABTS and FRAP methods (R^2^ = 0.77 for DPPH, 0.71 for ABTS and 0.73 for FRAP).

The influence of polyphenolic compounds on antioxidant capacity has been repeatedly described in the literature. The results clearly show that polyphenols play a significant role in shaping antioxidant capacity. Their power to scavenge free radicals depends on their structure and the group to which they belong [33,34,35,36]. These results agree with the study presented by Faraq et al. [20] who analyzed the antioxidant effect of *O. ficus-indica* in the crude extracts of pulps and peels. They showed the highest in vitro antioxidant activity in extracts with the highest total phenolic content, when tested using ABTS and DPPH assays [20]. These data were also confirmed by Chougui et al. [8].

### 2.4. Protein and Amino Acid Composition

The protein content was influenced by the variety of prickly pear (Table 4).

The “Fresa” and “Nopal ovalado” varieties were characterized by the significantly highest protein content (9.97 g/100 g DM), as compared to the “Orito” variety where this value was the lowest (7.09 g/100 g DM). Several studies have reported that prickly pear seeds are considered a nontraditional source of protein [14,37,38] and the protein content found in these studies was higher compared to the present data. Özcan and Juhaimi [11] and El Mannoubi et al. [39] found that the same seeds contain 4.78% crude protein. These differences may be influenced by growth conditions, variety, genetic factors, harvesting time, soil properties or geographical variations of prickly pear plants.

Analysing the amino acid composition of the protein contained in prickly pear seeds (*O. ficus-indica*), it was found that the variety had a significant effect on the content of individual amino acids and their sum in the tested samples (Table 5). 

Protein from the prickly pear seeds of the “Nopal alargado” variety contained the highest values for total indispensable amino acids (IAAs) and total dispensable amino acids (DAAs)—21.60 and 47.36 g/100 g, respectively, while the “Fresa” variety was characterized by the lowest total IAA and DAA content—10.30 and 22.90 g/100 g, respectively. Protein from “Nopal tradicional”, “Nopal ovalado”, “Orito” and “Nalle” prickly pear varieties was characterized by a similar content of total IAAs and DAAs, on average 18.97 and 43.58 g/100 g, respectively. Glutamic acid was the predominant amino acid followed by arginine, aspartic acid and leucine, independent of prickly pear variety. These results are in agreement with the study presented by Nassar [38] who analyzed the chemical composition and functional properties of prickly pear seed flour and its protein concentrate. However, a higher total IAA content and therefore higher IAA/DAA ratio (0.65) was noted in comparison to the present study, where this value was found to be on average 0.44, independent of prickly pear variety. These data were also confirmed by Sawaya et al. [40].

The value of the proteins derived from the seeds is determined by the presence of a set of amino acids, including all exogenous amino acids, i.e., lysine, methionine, tryptophan, threonine, valine, leucine, isoleucine and phenylalanine, and the relatively exogenous histidine. However, the most important in nutrition are lysine, sulphur amino acids, threonine, tryptophan, valine and isoleucine. The quality of the protein in the tested seeds was evaluated according to its content of IAAs in comparison to the reference protein pattern of FAO/WHO [41], as shown in Table 5. From the data obtained, it can be observed that none of the tested protein from prickly pear seeds of different varieties contained an adequate amount of all IAAs. In “Nopal tradicional”, “Nopal alargado”, “Nopal ovalado”, “Nopal espinoso”, “Orito” and “Nalle” prickly pear varieties, the first limiting amino acid was lysine and the second and third were methionine and cysteine, except for “Fresa” seeds where an inverse relationship was observed. This means that protein from prickly pear seeds is incomplete protein. On the other hand, Sawaya et al. [40] stated that prickly pear protein is a significantly good source of the sulphur amino acids (Met + Cys), which are generally the most limiting amino acids in seed proteins. In this respect, prickly pear seed protein is comparable to sesame protein which is high in sulphur-containing amino acids, containing about 6 g of methionine and cysteine/100 g.

### 2.5. Fat and Fatty Acid Composition

Table 6 shows the composition of the fatty acids in the fat extracted from the prickly pear seeds being analyzed.

The oil content obtained from the seven cultivars ranged from 2.61% for “Nalle” to 7.69% for “Nopal ovalado” (Table 6). De Wit et al. [9] obtained slightly higher values, ranging from 4.09% to 8.76% for 42 cultivars from South Africa, while those obtained by Labuschagne and Hugo [42] were slightly lower—from 2.24% to 5.59%. These differences may be due to growth conditions, cultivar and genetic factors as well as harvesting time, degree of ripeness or fruit processing [9]. The oil was mainly composed of unsaturated fatty acids, including polyunsaturated fatty acids (PUFA), that is, linoleic acid, and monounsaturated fatty acids (MUFA), mostly oleic acid, with a lower but significant fraction of saturated acids (SFA). Overall, 13 different fatty acids were identified and assessed. The dominant fatty acid was linoleic acid with a content varying between 57.72% (“Nopal ovalado”) and 63.11% (“Nopal espinoso”). Linoleic acid (*n*−3) was detected at a concentration lower than 1%, with the exception of “Nopal tradicional” and “Nopal espinoso” varieties. The highest PUFA content was measured in the variety “Nopal espinoso” (64.33%), and the lowest in “Nopal ovalado” (58.74%). Similar levels of PUFA were observed by Cirimina at al. [43] in oil extracted from Sicilian varieties. Among the MUFA, oleic acid occurred in the greatest amounts, from 19.37% (in “Nopal espinoso”) to 21.79% (in “Nopal tradicional”). Although there was a slight difference in MUFA content between the varieties analyzed, the average MUFA content was highest in the variety “Nopal tradicional”. Two dominant saturated fatty acids were palmitic acid with the share between 12.47% (in “Nopal espinoso”) to 15.06% (in “Nopal alargado”) and stearic acid, which varies from 2.56% (in “Nopal espinoso”) to 4.10% (in “Nalle”). The obtained results are in accordance with those of other researchers. Observed differences between analyzed cultivars could be connected with genetic factors.

## 3. Materials and Methods

### 3.1. Reagents and Standards

Acetonitrile, formic acid, methanol, DPPH (1,1-diphenyl-2 picrylhydrazyl radical), Trolox (6-hydroxy-2,5,7,8-tetramethylchroman-2-carboxylic acid), TPTZ [2,4,6-tri(2-pyridyl)-s-triazine], caffeic acid and boron trifluoride in methanol were purchased from Sigma-Aldrich (Steinheim, Germany). Quercetin 3-*O*-galactoside, isorhamnetin 3-*O*-glucoside and ferulic acid were purchased from Extrasynthese (Lyon, France). Diethyl ether was purchased from Chempur (Piekary Śląskie, Poland). Ninhydrine, hydrantine, methylcellosolve and sodium acetate buffer were purchased from Ingos company (Prague, Czech Republic).

### 3.2. Plant Material and Sample Processing

Prickly pear fruits from “Nopal alargado”, “Nopal espinoso”, “Nopal ovalado” and “Nopal tradicional” cultivars were collected at the experimental field station of Miguel Hernández University, in the province of Alicante, Spain (02°03′50″ E, 38°03′50″ N, and 25 masl). Another three cultivars were collected from private farms of Murcia (“Fresa” cultivar) and Alicante (“Nalle” and “Orito” cultivars). Plant species were identified by an expert botanist from the Department of Plant Sciences and Microbiology, using the protocol by García-Rollán [44].

The harvest of the fruits was done during the summer of 2018 and 2019. Fruits were manually picked at the same ripening stage, and immediately transported to the laboratory. In this way, a total of 30 fruits per cultivar and year were collected. One voucher of each cultivar is kept in the Miguel Hernández University herbarium (#152019). Table 7 presents the characteristics of the analyzed prickly pear cultivars.

Once in the laboratory, the spines of fruits were removed with a brush under tap water for 2 min, peeled, the fruits were cut into small pieces and submerged in water for a week to make the removal of the pulp easier. After this time, the water was removed, and the seeds were washed under tap water for two minutes to remove the pulp completely. After that, the seeds were placed on blotting paper and were left to dry at room temperature for ten days, and frozen at −80 °C until the time of analysis.

### 3.3. Identification and Quantification of Polyphenols by the UPLC-PDA-MS Method

For the extraction and determination of polyphenols, a protocol described before by Kolniak-Ostek [45] was followed.

Identification of polyphenols of prickly pear extracts was carried out using an ACQUITY Ultra Performance LC system equipped with a photodiode array detector with a binary solvent manager (Waters Corporation, Milford, MA, USA) with a mass detector G2 Q-Tof micromass spectrometer (Waters, Manchester, UK) equipped with an electrospray ionization (ESI) source operating in negative mode. The separation of individual polyphenols was carried out using a UPLC BEH C18 column (1.7 mm, 2.1 × 100 mm, Waters) at 30 °C.

The samples (10 µL) were injected, and the elution was completed in 15 min with a sequence of linear gradients and constant flow rates of 0.42 mL/min. The mobile phase consisted of solvent A (0.1% formic acid, *v*/*v*) and solvent B (100% acetonitrile). The linear gradient was as follows: 0.0–1.0 min, 99% A, 0.42 mL/min (isocratic), 1.0–12.0 min, 65.0% A, 0.42 mL/min (linear), 12.0–12.5 min, 99% A, 0.42 mL/min (linear), 12.5–13.5 min, 99% A, 0.42 mL/min (isocratic). The analysis was carried out using full-scan, data-dependent MS scanning from *m*/*z* 100–1500. Leucine enkephalin was used as the reference compound at a concentration of 500 pg/mL, and the [M − H]¯ ion at 554.2615 Da was detected. The [M − H]¯ ions were detected during a 15 min analysis performed within ESI–MS accurate mass experiments, which were permanently introduced via the LockSpray channel using a Hamilton pump. The lock mass correction was ±1.000 for the mass window. The mass spectrometer was operated in negative-ion mode, set to the base peak intensity (BPI) chromatograms, and scaled to 12,400 counts per second (cps) (100%). The optimized MS conditions were as follows: capillary voltage of 2500 V, cone voltage of 30 V, source temperature of 100 C, desolvation temperature of 300 °C, and desolvation gas (nitrogen) flow rate of 300 L/h.

Collision-induced fragmentation experiments were performed using argon as the collision gas, with voltage ramping cycles from 0.3 to 2 V. Characterization of the single components was carried out via the retention time and the accurate molecular masses. Each compound was optimized to its estimated molecular mass in the negative mode, before and after fragmentation. The data obtained from UPLC–MS were subsequently entered into the MassLynx 4.0 ChromaLynx Application Manager software (Waters).

The runs were monitored at the following wavelengths: phenolic acids at 320 nm and flavonol glycosides at 360 nm. The PDA spectra were measured over the wavelength range of 200–600 nm in steps of 2 nm. The retention times and spectra were compared to those of the authentic standards.

The quantification of phenolic compounds was performed by external calibration curves (R^2^ > 0.999), using reference compounds selected based on the principle of structure-related target analyte/standard (chemical structure or functional group). Standard stock solutions were diluted to appropriate concentrations (five calibration points were used in each case) for the plotting of calibration curves. The linearity was obtained by plotting the peak areas versus the corresponding concentrations (ppm) of each analyte. The calibration curve for caffeic acid was used to quantify caffeic acid hexosides. The calibration curve of ferulic acid was used to quantify ferulic acid derivatives. Protocatechuic acid hexoside was quantified with protocatechuic acid calibration curve.

The calibration curves of quercetin, quercetin rutinoside, and 3-*O*-galactoside were used to quantify quercetin derivatives. For isorhamnetin quantification, isorhamnetin 3-*O*-rutinoside and 3-*O*-glucoside were used.

All determinations were done in triplicate (*n* = 3). The results were expressed as milligrams per kg of dry matter (DM).

### 3.4. Antioxidant Capacity

The total in vitro antioxidant potential of samples was determined using a ferric reducing ability of plasma (FRAP) assay by Benzie and Strain [46] as a measure of antioxidant power. The DPPH and ABTS radical scavenging activities of samples were determined according to the method of Yen and Chen [47] and Re et al. [48]. The powder samples (0.5 g) were extracted with 10 mL of 80% methanol acidified with 1% HCl (*v*/*v*). The extraction was performed by incubation for 20 min under sonication (300 W, 40 kHz; Sonic 6D, Polsonic, Warsaw, Poland) with occasional shaking. This method has proved to be adequate for complete extraction. Next, the slurry was centrifuged at 19,000 g for 10 min, and the supernatant was filtered through a hydrophilic PTFE 0.20 µm membrane (Millex Samplicity Filter, Merck) and used for analysis A standard curve was prepared using different concentrations of Trolox. All determinations were performed in triplicate using a Shimadzu UV-2401 PC spectrophotometer (Kyoto, Japan). The results were corrected for dilution and expressed in μmol Trolox Equivalent per kg of DM.

### 3.5. Proximate Composition

The total protein content was evaluated according to the Kjeldahl method of the Association of Analytical Chemists [49]. Approximately 1 g of raw material was hydrolyzed with 25 mL concentrated sulfuric acid (H_2_SO_4_) containing one catalyst tablet in a heat block (Büchi Digestion Unit K-424, Labortechnik AG, Flawil, Switzerland) at 370 °C for 2 h. After cooling, H2O was added to the hydrolysates before neutralization, using a Büchi Distillation Unit K-355 (Athens, Greece) and titration. A nitrogen to protein conversion factor of 6.25 was used to calculate total protein. Fat content was determined according to the standard method of the Association of Official Analytical Chemists International [50]. A sample of 2 g of ground seeds was hydrolyzed using 4N HCl. Fat extraction and solvent (diethyl ether) removal were performed in an automated Soxhlet apparatus B-811 (Büchi Labortechnik AG, Flawil, Switzerland); the extraction time was 180 min.

### 3.6. Amino Acid Analysis

The amino acid composition of prickly pear seeds was determined by ion-exchange chromatography after 23 h’ hydrolysis with 6 N HCl at 110 °C. After cooling, filtering and washing, the hydrolyzed sample was evaporated in a vacuum evaporator at a temperature below 50 °C. The dry residue was dissolved in a buffer of pH 2.2. The prepared sample was analyzed using the ninhydrin method [51,52]. The pH 2.6, 3.0, 4.25, and 7.9 buffers were applied. The ninhydrin solution was buffered at pH 5.5. The hydrolyzed amino acids were determined using an AAA-400 analyzer (INGOS, Prague, Czech Republic). A photometric detector was used, working at two wavelengths, 440 nm and 570 nm. A column of 350 × 3.7 mm, packed with ion exchanger Ostion LG ANB (INGOS) was utilized. Column temperature was kept at 60−74 °C and the detector at 121 °C. The calculations were carried out relative to an external standard. No analysis of tryptophan was carried out.

### 3.7. Quantitative Evaluation of Protein Quality

The amino acid content in opuntia seeds was expressed on the nitrogen basis (g per 16 g N) and it was compared to a reference protein. The amino acid pattern for high-quality protein established by the Joint Food and Agriculture Organisation/World Health Organisation (FAO/WHO) Committee in 1991. Levels were calculated on the basis of the essential amino acid composition of the chemical scores (CS), according to the Mitchell and Block method [53] and the integrated EAA index [54].

### 3.8. Fatty Acids Analysis

Fatty acid composition of seeds oil was determined by GC, according to the American Oil Chemists’ Society Official Method Ce 1-62 [55]. Boron trifluoride in methanol was used as methylating agent. Fatty acid methyl esters (FAMEs) were analyzed by an Agilent 7820A gas chromatograph (Agilent Technologies, Santa Clara, CA, USA), equipped with a capillary column RTX-2330, 105 m length, 0.25 mm i.d., 0.2 μm film thickness (Restek, Bellefonte, PA, USA). Injector and detector (FID) temperatures were 260 °C and 280 °C, respectively. Column temperature was set to 200 °C for 21 min, then increased to 250 °C at a rate of 10 °C/min; the final temperature was held for 6 min. Helium was used as a carrier gas, at a linear flow rate of 35 cm/sec. Individual FAMEs were identified using the Certified Reference Material (CRM) 47885 (Supelco, Bellefonte, PA, USA). The following fatty acid combinations were calculated: total saturate fatty acids (SFA), total monounsaturated fatty acids (MUFA) and total polyunsaturated fatty acids (PUFA).

## 4. Conclusions

The research conducted has shown that the seeds of the prickly pear (*O. ficus-indica*) are an excellent source of nutrients and health-promoting substances. Due to the high content of phenolic compounds, they are characterized by strong antioxidant properties. The seeds of the prickly pear are usually discarded after extraction of pulp, providing a large amount of seeds as waste. Prickly pear seeds can be used as a low-cost source of health-promoting compounds. Additionally, this would contribute to reducing the amount of waste generated during the production process.

## Figures and Tables

**Table 1:** Retention times, UV–vis spectra and characteristic ions of phenolic compounds and organic acids of the “Orito” cultivar.

Peak No.	Rt	UV [nm]	MS [M − H]^−^ (*m/z*) ^a^	MS/MS (*m/z*) ^a^	Tentative Identification
1.	0.62	360	195.0522	177.0404; 159.2073; 129.0194	Gluconic acid
2.	1.66	210	191.0051	110.9974	(Iso)Citric acid
3.	2.04	340	301.0920	179.0521; 151.0274	Quercetin
4.	2.62	220, 275	255.0366	237.0222; 193.0362; 165.0417	Piscidic acid
5.	3.38	299	315.0781	153.0173	Protocatechuic acid hexoside
6.	3.68	325	517.1523	386.1084; 193.0476	Ferulic acid derivative
7.	4.39	220, 275	239.0416	179.0218; 149.0408	Eucomic acid
8.	5.02	325	341.0837	179.0372	Caffeic acid glucoside I
9.	5.18	325	517.1543	193.0480; 175.0378	Ferulic acid diglucoside
10.	5.94	324	341.0697	179.0523	Caffeic acid glucoside II
11.	6.40	325	371.1151	249.9247; 193.0503; 175.0273	Feruloyl gluconic acid
12.	6.73	202, 216, 275	565.1764	339.1087; 327.1086	-
13.	6.91	325	595.2080	329.1040; 197.8092; 175.0987; 193.0476; 162.8393	Ferulic acid derivative
14.	7.49	350	609.1295	301.0355	Quercetin-3-O-rutinoside
15.	7.69	355	463.1399	301.0355	Quercetin-3-O-galactoside
16.	7.75	350	755.1174	623.0457; 315.0475	Isorhamnetin-pentosyl rutinoside
17.	8.04	340	593.1521	315.0511	Isorhamnetin-pentosyl rhamnoside
18.	8.28	350	623.1598	315.0420	Isorhamnetin-3-O-rutinoside
19.	8.91	350	477.1020	315.0455	Isorhamnetin-3-O-galactoside
20.	8.96	350	477.1022	315.0475	Isorhamnetin-3-O-glucoside
21.	9.41	340	519.1130	315.0510	Isorhamnetin acylated hexoside

^a^ Experimental data.

**Table 2:** Phenolic acid and flavonol composition of different varieties of prickly pear (mg/kg of dry matter) *.

Peak No.	[mg/kg]	Fresa	Nopal Alargado	Nopal Espinoso	Nalle	Nopal Ovalado	Nopal Tradicional	Orito
	*Phenolic acids*							
5.	Protocatechuic acid hexoside	4.57 ± 0.2 ^a^	7.13 ± 0.2 ^b^	22.36 ± 1.1 ^f^	11.37 ± 0.2 ^c^	11.24 ± 0.2 ^c^	12.56 ± 0.1 ^d^	19.03 ± 0.3 ^e^
6.	Ferulic acid derivative	2.35 ± 0.1 ^c^	0.00 ± 0.0 ^a^	0.00 ± 0.0 ^a^	11.62 ± 0.1 ^d^	1.08 ± 0.0 ^b^	0.00 ± 0.0 ^a^	0.00 ± 0.0 ^a^
8.	Caffeic acid hexoside I	1.28 ± 0.0 ^a^	4.35 ± 0.3 ^b^	12.59 ± 0.2 ^e^	11.95 ± 0.2 ^d^	4.09 ± 0.1 ^b^	5.51 ± 0.1 ^c^	13.50 ± 0.9 ^f^
9.	Ferulic acid diglucoside	4.10 ± 0.1 ^a^	37.07 ± 1.2 ^c^	108.97 ± 3.5 ^e^	63.46 ± 3.3 ^d^	13.59 ± 1.0 ^b^	31.68 ± 1.2 ^c^	60.56 ± 2.2 ^d^
10.	Caffeic acid hexoside II	1.75 ± 0.0 ^c^	2.41 ± 0.1 ^d^	1.54 ± 0.0 ^b^	1.07 ± 0.0 ^a^	2.40 ± 0.0 ^d^	2.61 ± 0.0 ^d^	1.71 ± 0.0 ^c^
11.	Feruloyl gluconic acid	2.64 ± 0.0 ^a^	2.43 ± 0.1 ^a^	14.21 ± 0.5 ^c^	3.67 ± 0.0 ^b^	2.20 ± 0.1 ^a^	3.22 ± 0.0 ^b^	2.57 ± 0.1 ^a^
13.	Ferulic acid derivative	2.35 ± 0.1 ^a^	7.09 ± 0.3 ^d^	11.93 ± 0.2 ^f^	6.28 ± 0.5 ^c^	4.69 ± 0.2 ^b^	10.16 ± 0.3	7.64 ± 0.4 ^e^
	Sum of phenolic acids	19.05	60.47	171.60	109.44	39.29	65.75	105.01
	*Flavonols*							
3.	Quercetin	1.95 ± 0.0 ^d^	1.21 ± 0.0 ^b^	0.90 ± 0.0 ^a^	8.40 ± 0.4 ^e^	1.52 ± 0.1 ^c^	2.03 ± 0.0 ^d^	1.16 ± 0.1 ^ab^
14.	Quercetin-3-*O*-rutinoside	0.96 ± 0.0 ^a^	5.95 ± 0.3 ^c^	8.34 ± 0.2 ^e^	5.41 ± 0.1 ^c^	4.18 ± 0.1 ^b^	7.48 ± 0.6 ^d^	4.78 ± 0.1 ^b^
15.	Quercetin-3-*O*-galactoside	1.46 ± 0.1 ^a^	4.99 ± 0.2 ^e^	3.58 ± 0.1 ^c^	4.96 ± 0.1 ^e^	2.62 ± 0.0 ^b^	7.13 ± 0.3 ^f^	4.34 ± 0.3 ^d^
16.	Isorhamnetin-pentosyl rutinoside	1.97 ± 0.1 ^a^	2.98 ± 0.2 ^b^	3.71 ± 0.1 ^c^	1.60 ± 0.0 ^a^	3.92 ± 0.1 ^c^	4.77 ± 0.0 ^d^	1.67 ± 0.1 ^a^
17.	Isorhamnetin-pentosyl rhamnoside	1.05 ± 0.0 ^bc^	1.23 ± 0.1 ^c^	0.86 ± 0.0 ^b^	0.94 ± 0.0 ^b^	0.12 ± 0.0 ^a^	0.15 ± 0.0 ^a^	1.03 ± 0.0 ^bc^
18.	Isorhamnetin-3-*O*-rutinoside	1.23 ± 0.0 ^b^	1.62 ± 0.0 ^c^	1.59 ± 0.0 ^c^	1.10 ± 0.0 ^b^	0.64 ± 0.0 ^a^	1.82 ± 0.0 ^de^	1.94 ± 0.0 ^e^
19.	Isorhamnetin-3-*O*-galactoside	0.00 ± 0.0 ^a^	2.06 ± 0.1 ^e^	3.31 ± 0.1 ^f^	1.11 ± 0.0 ^c^	0.61 ± 0.0 ^b^	1.99 ± 0.1 ^e^	1.66 ± 0.0 ^d^
20.	Isorhamnetin-3-*O*-glucoside	2.65 ± 0.2 ^a^	38.92 ± 1.1 ^d^	51.03 ± 1.6 ^e^	22.76 ± 0.8 ^c^	12.75 ± 0.6 ^b^	29.81 ± 1.1 ^cd^	23.93 ± 1.0 ^c^
21.	Isorhamnetin acylated hexoside	3.75 ± 0.2 ^a^	17.26 ± 1.0 ^c^	21.74 ± 1.1 ^d^	21.21 ± 0.6 ^d^	3.46 ± 0.1 ^a^	10.28 ± 0.6 ^b^	17.69 ± 1.1 ^c^
	Sum of flavonols	15.02	76.22	95.07	67.49	29.83	65.47	58.21
	Total	34.07	136.69	266.67	176.92	69.13	131.21	163.22

* Values are means ± standard deviation. *n* = 6; Amounts of phenolic acids and flavonols were converted into caffeic acid (caffeic acid derivatives), protocatechuic acid (protocatechuic acid derivatives), ferulic acid (ferulic acid derivatives), quercetin 3-*O*-glucoside (quercetin derivatives), isorhamnetin 3-*O*-glucoside (isorhamnetin derivatives); ^a–f^ the same letters within the same row were not significantly different.

**Table 3:** In vitro antioxidant capacity of different varieties of prickly pear (mMol Trolox/kg of dry matter) *.

[mMol Trolox/kg]	Fresa	Nopal Alargado	Nopal Espinoso	Nalle	Nopal Ovalado	Nopal Tradicional	Orito
DPPH	1.39 ± 0.0 ^a^	2.62 ± 0.0 ^b^	4.99 ± 0.0 ^d^	3.11 ± 0.0 ^c^	2.57 ± 0.0 ^b^	1.92 ± 0.0 ^a^	2.44 ± 0.0 ^b^
ABTS	7.08 ± 0.1 ^a^	10.43 ± 0.2 ^c^	11.67 ± 0.2 ^e^	11.33 ± 0.1 ^d^	9.97 ± 0.1 ^b^	10.04 ± 0.1 ^b,c^	11.49 ± 0.1 ^d^
FRAP	3.67 ± 0.0 ^a^	6.14 ± 0.0 ^c^	8.89 ± 0.1 ^f^	6.77 ± 0.0 ^d^	6.86 ± 0.1 ^d^	5.9 ± 0.0 ^b^	7.22 ± 0.1 ^e^

* Values are means ± standard deviation. *n* = 6; ^a–f^ the same letters within the same row were not significantly different.

**Table 4:** Fat and protein content of seeds of different varieties of prickly pear (g/100 g dry matter) *.

[g/100 g]	Fresa	Nopal Alargado	Nopal Espinoso	Nalle	Nopal Ovalado	Nopal Tradicional	Orito
Protein	9.97 ± 0.5 ^f^	9.45 ± 0.2 ^d^	9.61 ± 0.2 ^e^	6.36 ± 0.2 ^a^	9.97 ± 0.3 ^f^	7.69 ± 0.4 ^c^	7.09 ± 0.1 ^b^
Fat	4.94 ± 0.2 ^b^	6.17 ± 0.3 ^d^	5.24 ± 0.3 ^c^	2.61 ± 0.1 ^a^	3.25 ± 0.2 ^c^	5.97 ± 0.3 ^d^	4.39 ± 0.2 ^c^

* Values are means ± standard deviation. *n* = 6; ^a–f^ the same letters within the same row were not significantly different.

**Table 5:** Amino acid concentration in different varieties of prickly pear seeds (g/100 g of protein) *.

Amino Acids	Fresa	Nopal Alargado	Nopal Espinoso	Nalle	Nopal Ovalado	Nopal Tradicional	Orito	FAO/WHO Reference Pattern (1991)
IAA *								
LEU	2.41 ± 0.04 ^b^	4.75 ± 0.08 ^a^	3.55 ± 0.28 ^a,b^	4.23 ± 0.11 ^a^	4.04 ± 0.43 ^a,b^	4.05 ± 0.35 ^a,b^	4.24 ± 0.19 ^a^	6.60
Leucine	1.33 ± 0.03 ^b^	2.54 ± 0.04 ^a^	1.98 ± 0.12 ^a,b^	2.45 ± 0.18 ^a^	2.24 ± 0.24 ^a^	2.24 ± 0.15 ^a^	2.32 ± 0.12 ^a^	2.80
Isoleucine	0.32 ± 0.02 ^b^	0.56 ± 0.02 ^b^	0.72 ± 0.21 ^a^	0.63 ± 0.07 ^a,b^	0.78 ± 0.14 ^a^	0.65 ± 0.17 ^a,b^	0.64 ± 0.09 ^a,b^	
Methionine	0.22 ± 0.01 ^b^	0.58 ± 0.05 ^a^	0.44 ± 0.06 ^a,b^	0.58 ± 0.04 ^a^	0.46 ± 0.11 ^a^	0.60 ± 0.06 ^a^	0.49 ± 0.05 ^a^	
Cysteine	0.54	1.14	1.16	1.21	1.24	1.25	1.13	2.50
Cysteine+Methionie	1.32 ± 0.04 ^b^	3.13 ± 0.09 ^a^	2.14 ± 0.18 ^a,b^	2.57 ± 0.10 ^a^	2.53 ± 0.34 ^a^	2.54 ± 0.29 ^a^	2.53 ± 0.11 ^a^	
Phenyloalanie	1.08 ± 0.08 ^b^	2.39 ± 0.09 ^a^	1.83 ± 0.11 ^a,b^	2.06 ± 0.02 ^a^	2.17 ± 0.29 ^a^	2.08 ± 0.16 ^a^	2.08 ± 0.05 ^a^	3.40
Threonine	1.93	4.77	3.28	4.63	3.82	4.08	3.89	6.30
Phenyloalanine+Threonine	1.48 ± 0.05 ^b^	2.59 ± 0.04 ^a^	2.09 ± 0.17 ^a,b^	2.41 ± 0.04 ^a,b^	2.43 ± 0.25 ^a,b^	2.32 ± 0.21 ^a,b^	2.39 ± 0.09 ^a,b^	5.80
Lysine	0.61 ± 0.03 ^b^	1.64 ± 0.10 ^a^	1.14 ± 0.11 ^a,b^	1.42 ± 0.14 ^a^	1.29 ± 0.25 ^a,b^	1.54 ± 0.19 ^a^	1.36 ± 0.09 ^a,b^	
Tyrosine	1.53 ± 0.25 ^b^	3.43 ± 0.06 ^a^	2.60 ± 0.21 ^a,b^	2.77 ± 0.15 ^a^	3.00 ± 0.31 ^a^	2.94 ± 0.10 ^a^	2.82 ± 0.10 ^a^	3.50
Valine	10.30	21.60	16.49	19.12	18.94	18.97	18.86	
DAA **								
ASP	2.78 ± 0.23 ^c^	5.82 ± 0.08 ^a^	4.42 ± 0.37 ^a,b^	5.16 ± 0.54 ^a^	5.33 ± 0.74 ^a^	4.99 ± 0.40 ^a,b^	5.05 ± 0.16 ^a,b^	
Aspartic acid	5.44 ± 0.41 ^b^	13.85 ± 0.23 ^a^	9.69 ± 1.33 ^a,b^	12.02 ± 0.33 ^a^	11.44 ± 1.73 ^a,b^	11.83 ± 1.13 ^a^	11.40 ± 0.36 ^a,b^	
Glutamic acid	1.40 ± 0.10 ^b^	2.76 ± 0.07 ^a^	2.40 ± 0.20 ^a,b^	2.66 ± 0.22 ^a^	2.74 ± 0.35 ^a^	2.55 ± 0.18 ^a^	2.51 ± 0.08 ^a,b^	
Serine	1.97 ± 0.13 ^b^	4.79 ± 0.17 ^a^	3.66 ± 0.46 ^a,b^	4.13 ± 0.11 ^a^	3.65 ± 0.43 ^a,b^	4.18 ± 0.42 ^a^	4.27 ± 0.17 ^a^	
Glycine	2.14 ± 0.03 ^b^	3.65 ± 0.05 ^a^	3.18 ± 0.21 ^a,b^	3.48 ± 0.45 ^a^	4.02 ± 0.27 ^a^	3.71 ± 0.23 ^a^	3.21 ± 0.21 ^a,b^	
Alanine	1.44 ± 0.39	2.77 ± 0.53	2.43 ± 0.12	2.66 ± 0.12	2.31 ± 0.22	2.67 ± 0.57	2.63 ± 0.58	
Histidine	4.21 ± 0.04 ^c^	9.58 ± 0.52 ^a^	6.09 ± 0.67 ^a,b^	8.08 ± 0.43 ^a,b^	7.62 ± 1.03 ^a,b^	8.17 ± 0.85 ^a,b^	7.93 ± 0.42 ^a,b^	
Arginine	3.52 ± 0.22 ^b^	4.15 ± 0.17 ^a,b^	7.39 ± 0.32 ^a^	6.89 ± 0.18 ^a,b^	7.09 ± 0.46 ^a,b^	5.18 ± 0.16 ^a,b^	4.71 ± 0.28 ^a,b^	
Proline	22.90	47.36	39.25	45.08	44.20	43.28	41.75	
IAA/DAA	0.45	0.46	0.42	0.42	0.43	0.44	0.45	
I limiting amino acid	Met+Cys	Lys	Lys	Lys	Lys	Lys	Lys	
II limiting amino acid	Lys	Met+Cys	Met+Cys	Met+Cys	Met+Cys	Met+Cys	Met+Cys	
TP	9.97 ± 0.5 ^a^	9.45 ± 0.2 ^c^	9.61 ± 0.2 ^b^	6.36 ± 0.2 ^f^	9.97 ± 0.3 ^a^	7.69 ± 0.4 ^d^	7.09 ± 0.1 ^e^	

* Values are means ± standard deviation. *n* = 4; ^a–f^ the same letters within the same row were not significantly different; * IAA-indispensable amino acids; ** DAA-dispensable amino acids.

**Table 6:** Fat content (%) and fatty acid composition (% of total fatty acid profile) of prickly pear seeds as affected by cultivar *.

	Fresa	Nopal Alargado	Nopal Espinoso	Nalle	Nopal Ovalado	Nopal Tradicional	Orito
Fat content (%)	4.99 ± 0.2 ^b^	6.17 ± 0.3 ^d^	5.24 ± 0.3 ^c^	2.61 ± 0.1 ^a^	7.69 ± 0.2 ^e^	5.97 ± 0.3 ^d^	5.55 ± 0.2 ^c^
Fatty acid (%)							
Miristic acid (C 14:0)	0.01 ± 0.01 ^a^	0.02 ± 0.01 ^a^	0.01 ± 0.01 ^a^	0.01 ± 0.01 ^a^	0.01 ± 0.01 ^a^	0.01 ± 0.01 ^a^	0.02 ± 0.01 ^a^
Palmitic acid (C 16:0)	14.56 ± 0.53 ^d^	15.06 ± 0.35 ^e^	12.47 ± 0.41 ^a^	13.83 ± 0.28 ^b^	14.33 ± 0.26 ^c^	13.77 ± 0.82 ^b^	14.16 ± 0.51 ^c^
Palmitooleic acid (C 16:1)	0.82 ± 0.28 ^a^	0.79 ± 0.12 ^a^	0.78 ± 0.12 ^a^	0.84 ± 0.15 ^a^	0.85 ± 0.25 ^a^	0.91 ± 0.32 ^a^	0.83 ± 0.30 ^a^
Margaric acid (C 17:0)	0.02 ± 0.01 ^a^	0.01 ± 0.00 ^a^	0.03 ± 0.01 ^a^	0.01 ± 0.01 ^a^	0.02 ± 0.01 ^a^	0.01 ± 0.00 ^a^	0.02 ± 0.01 ^a^
Stearic acid (C 18:0)	3.50 ± 0.22 ^b^	3.36 ± 0.17 ^b^	2.56 ± 0.21 ^a^	4.12 ± 0.16 ^c^	4.00 ± 0.27 ^c^	3.62 ± 0.31 ^b^	3.88 ± 0.28 ^c^
Oleic acid (C 18:1)	20.26 ± 0.61 ^b^	20.48 ± 0.51 ^b^	19.37 ± 0.37 ^a^	21.23 ± 0.41 ^d^	21.64 ± 0.41 ^e^	21.79 ± 0.54 ^e^	20.77 ± 0.41 ^c^
Liloleic acid (C 18:2)	60.04 ± 0.43 ^d^	59.38 ± 0.66 ^c^	63.11 ± 0.82 ^e^	58.11 ± 0.48 ^c^	57.72 ± 0.70 ^a^	58.30 ± 0.72 ^b^	59.29 ± 0.55 ^c^
Linolenic acid (C 18:3)	0.23 ± 0.10 ^a^	0.33 ± 0.10 ^a^	1.10 ± 0.19 ^d^	0.54 ± 0.07 ^b^	0.89 ± 0.20 ^c^	1.01 ± 0.09 ^d^	0.46 ± 0.10 ^b^
Arachidic acid (C 20:0)	0.20 ± 0.01 ^a^	0.21 ± 0.01 ^a^	0.19 ± 0.03 ^a^	0.20 ± 0.02 ^a^	0.20 ± 0.02 ^a^	0.21 ± 0.02 ^a^	0.21 ± 0.02 ^a^
Gondoic acid (C 20:1)	0.09 ± 0.01 ^a^	0.11 ± 0.01 ^a^	0.11 ± 0.01 ^a^	0.09 ± 0.01 ^a^	0.12 ± 0.01 ^a^	0.10 ± 0.01 ^a^	0.12 ± 0.01 ^a^
Eicosatrienoic acid (C 20:3)	0.13 ± 0.02 ^a^	0.15 ± 0.01 ^a^	0.12 ± 0.01 ^a^	0.12 ± 0.01 ^a^	0.13 ± 0.02 ^a^	0.14 ± 0.01 ^a^	0.14 ± 0.01 ^a^
Behenic acid (C 22:0)	0.11 ± 0.01 ^a^	0.09 ± 0.01 ^a^	0.12 ± 0.01 ^a^	0.10 ± 0.01 ^a^	0.08 ± 0.01 ^a^	0.10 ± 0.10 ^a^	0.09 ± 0.01 ^a^
Lignoceric acid (C 24:0)	0.03 ± 0.02 ^a^	0.01 ± 0.01 ^a^	0.03 ± 0.01 ^a^	0.01 ± 0.01 ^a^	0.01 ± 0.01 ^a^	0.02 ± 0.01 ^a^	0.01 ± 0.01 ^a^
∑ MUFA	21.17 ± 0.6 ^b^	21.38 ± 0.33 ^b^	20.26 ± 0.32 ^a^	22.16 ± 0.34 ^d^	22.61 ± 0.29 ^e^	22.71 ± 0.55 ^e^	21.72 ± 0.41 ^c^
∑ PUFA	60.40 ± 0.38 ^d^	59.86 ± 0.51 ^c^	64.33 ± 0.50 ^e^	59.56 ± 0.50 ^c^	58.74 ± 0.62 ^a^	59.55 ± 0.72 ^b^	59.89 ± 0.48 ^c^
∑ SFA	18.43 ± 0.34 ^d^	18.76 ± 0.36 ^e^	15.41 ± 0.31 ^a^	18.28 ± 0.17 ^c^	18.65 ± 0.45 ^e^	17.74 ± 0.21 ^b^	18.39 ± 0.25 ^d^

MUFA—monounsaturated fatty acids, PUFA—polyunsaturated fatty acids, SFA—saturated fatty acids. * Values are means ± standard deviation. *n* = 6; ^a–e^ the same letters within the same row were not significantly different.

**Table 7:** Characteristics of the analyzed prickly pear cultivars.

Cultivar	Characteristics
Fresa	Red cultivar. High amount of betalains and polyphenols. Weight of the fruit: 100–140 g.
Nalle	Green cultivar. Average weight of the fruit 90−100 g.
Nopal alargado	Green-yellow cultivar without prickles. Weight of the fruit: 120–160 g.
Nopal espinoso	Highly spiny green cultivar. Weight of the fruit 60–80 g.
Nopal ovalado	Green-yellow cultivar. Weight of the fruit: 90–120 g.
Nopal tradicional	Traditional cultivar (orange). Weight of the fruit: 90–120 g.
Orito	Orange cultivar. Average weight of the fruit 110−140 g. High fruit production.
